# Linkages between socioeconomic inequalities, pro-environmental behaviours, climate change concerns and experiences, and wellbeing outcomes in England

**DOI:** 10.1080/27658511.2025.2500182

**Published:** 2025-05-12

**Authors:** Jonathan R. Olsen, Claire L. Niedzwiedz, Ruth Dundas, Jala Rizeq, Gergő Baranyi, Srinivasa Vittal Katikireddi, Jill Pell

**Affiliations:** a MRC/CSO Social and Public Health Sciences Unit, School of Health and Wellbeing, University of Glasgow, Glasgow, UK; b School of Health and Wellbeing, University of Glasgow, Glasgow, UK; c Centre for Longitudinal Studies, UCL Institute of Education, University College London, London, UK

**Keywords:** Pro-environmental behaviour, climate worry, wellbeing, inequalities, flood, temperature, UK Household Longitudinal Study

## Abstract

The global health community recognises climate change as a public health emergency due to its direct and indirect impacts on health and wellbeing. This study explores sociodemographic differences in climate change concern and pro-environmental behaviours by socioeconomic status, their association with wellbeing, and whether experiences of climate change (e.g. residing in flood-affected or temperature-changing areas) mediate wellbeing outcomes. Using data from Understanding Society, a national panel survey in England (2018/19, *n* = 24,950, age 16+), the study examined climate concern, 11 pro-environmental behaviours, satisfaction with these behaviours, and three wellbeing outcomes: life satisfaction, optimism, and psychological distress. Data were spatially linked with flood (2010–18) and summer temperature changes (2001–2020). Climate concern varied by sociodemographic factors, with older and disadvantaged groups most satisfied with their behaviours. Individuals satisfied with their environmental actions reported better wellbeing, while dissatisfaction was linked to distress and worse life satisfaction. However, pro-environmental behaviours themselves were not associated with wellbeing. Residing in flood-affected or temperature-changing areas also showed no link to wellbeing. Addressing wellbeing impacts related to climate concern requires targeted mitigation strategies, especially for those dissatisfied with their environmental actions. Pro-environmental behaviours could act to mitigate against the potential adverse effects of eco-anxiety.

## 1. Introduction

The accelerated rise in global surface temperatures and the occurrence of extreme climate events, driven by the emission of greenhouse gases, present a formidable global threat (Lee et al., 2023). The global health community has officially recognised climate change as a public health emergency due to its immediate effects on health (Solomon & LaRocque, 2019). The repercussions of climate change on wellbeing are delineated through two main channels: (1) the ***direct impact*** on local communities as they endure extreme climatic events such as heatwaves, wildfires, or floods, and (2) the ***indirect impact***, leading to emotional distress, concern and anxiety about the future (Fritze et al., 2008; Manning & Clayton, 2018). While communities vary in their exposure to direct or indirect climate change events, both forms of exposure can negatively affect wellbeing (Lawrance et al., 2022). Many people are worried and concerned about the crisis (Hickman et al., 2021) and these negative psychological reactions could be considered appropriate given the scale of the climate crisis and may have motivational effects (Clayton, 2020). Climate change is believed to affect wellbeing through three primary pathways: the material conditions in which individuals live (including infrastructure, access to essential goods and services, and basic necessities like food and water); awareness of current or future climate impacts; and the effects of climate policies designed to promote adaptation or mitigation (Adger et al., 2022; Niedzwiedz et al., 2024). Some reactions can be functionally debilitating or distressing to people and are conceptualised within climate change anxiety (Clayton, 2020; Clayton & Karazsia, 2020) or eco-anxiety (Hogg et al., 2024). A growing body of evidence has highlighted the link between climate worry and wellbeing. For example, a meta-analysis of studies utilising the Climate Change Anxiety Scale found that higher levels of climate anxiety were associated with poorer wellbeing outcomes (Gago et al., 2024). Despite this, important gaps remain in understanding how individual-level factors contribute to pro-environmental behaviours and climate concern, as well as how different types of exposure to climate change—whether direct or indirect—shape wellbeing outcomes. Further research is needed to address these questions.

Evidence suggests that higher levels of individual pro-environmental behaviours may serve as a buffer against climate-related worry and adverse wellbeing outcomes. Studies show that greater climate change worry or concern is linked to pro-environmental actions (see review Ojala 2008). This connection can manifest in the form of seeking new information, reevaluating current behaviours, and a tendency towards pro-environmental behaviours (Pihkala, 2020; Whitmarsh et al., 2022). For example, a large multi-country study found that individuals engaging in more pro-environmental behaviours reported greater wellbeing, irrespective of socioeconomic factors such as income (Capstick et al., 2022). However, associations between climate events and specific wellbeing measures—including PTSD, anxiety, depression, life satisfaction, and subjective wellbeing—vary, emphasising the need for studies to carefully consider the wellbeing outcome, and consider a range of outcome measures (Capstick et al., 2022; Ogunbode et al., 2022; Waite et al., 2017).

Findings on the impact of experiencing previous climate events on wellbeing are inconsistent, often due to methodological differences across studies. For instance, a small UK study (*n* = 370) found no link between self-reported local flooding in the past 5 years and wellbeing of local residents (Ogunbode et al., 2022). In contrast, a study conducted after a significant UK flooding event a year earlier identified a pronounced and long-lasting negative impact on mental wellbeing, which extended beyond those directly affected by home flooding (Waite et al., 2017). This highlights the need for large-scale, comprehensive research examining the effects of climate events like flooding across broader geographical areas and over varying timeframes.

There is a suggestion that the influence of climate change is likely to intensify existing social and health inequalities in the UK. Notably, factors such as age, pre-existing medical conditions, and social deprivation have been identified as critical elements that heighten susceptibility to adverse wellbeing outcomes associated with climate change (Paavola, 2017), and research should seek to importance to understand the impacts across different population groups (Thomas et al., 2014). The pathways through which health inequalities may escalate due to climate change include adaptation strategies based on individual preparedness, selective uptake in action and behaviour change, and a lack of trust in public information messaging (Paavola, 2017). Additionally, factors like housing conditions, selective mobility, community cohesion, individual and community agency, institutional readiness, and perceptions of institutional readiness can have both direct and indirect impacts on health, exhibiting patterns that are both socially and geographically determined (Dodd et al., 2023). These factors cause deep rooted, complex socioeconomic social and health inequalities that national small area deprivation indices capture (Allik et al., 2020). Moreover, there is a lack of research investigating how sociodemographic factors influence climate concern, behaviours, and related wellbeing outcomes. Limited evidence exists on whether experiences of climate events, such as flooding or extreme heat, vary by sociodemographic characteristics and how such variability relates to wellbeing outcomes within nationally representative populations. Addressing these gaps is critical to understanding and mitigating the social and psychological impacts of climate change.

The study aims are to explore sociodemographic variation in climate change concern and behaviours by socioeconomic status, explore whether these are associated with wellbeing outcomes, if experience of climate change (residing in a flood effected or temperature changing area) varies by socioeconomic position, and if this mediates wellbeing outcomes. A number of confounders associated with life satisfaction, psychological distress, and optimism must also be considered in the pathways and subsequent analysis, these include age (Capone et al., 2021; López Ulloa et al., 2013), gender (Diener et al., 2018), employment status (Jebb et al., 2020), income (Capone et al., 2021; Diener et al., 2018), educational attainment (Kristoffersen, 2018), marital status (Jebb et al., 2020), general health (Diener et al., 2018), loneliness (VanderWeele et al., 2012), religious status (Diener et al., 2018), material deprivation (Kristoffersen, 2018), area-level socioeconomic deprivation, urbanicity and region (Hand, 2020).

### 1.2. Research questions

1. Explore variation in climate concern and behaviours by sociodemographic factors.
2. Explore whether individual-level environment concerns and behaviours associated with wellbeing outcomes (psychological distress, life satisfaction and optimism).
3. Explore whether experience of climate change (residing in a flood effected or temperature changing area) are associated with wellbeing outcomes.

## 2. Materials and methods

### 2.1. Study population

Analyses are based on data from *Understanding Society*, the UK Household Longitudinal Study (UKHLS) (University of Essex, 2022), details of which have been reported previously (Buck & McFall, 2011). The UKHLS, which began in 2009, is a panel survey of a stratified clustered random sample (Lynn, 2009) designed to be representative of the community-dwelling UK population. Data are currently available from 12 waves (Institute for Social and Economic Research, 2022). Questions relating to environmental issues were asked in wave 10 (2018–2019) and therefore the current analyses are based on adult respondents (aged 16+ years) who were interviewed in wave 10 with interview data linked to external climate and flood data. Special licence geographic identifiers data were provided for Understanding Society participants by Lower Layer Super Output Areas (LSOAs) in England (University of Essex, 2022). LSOAs are small geographical spatial areas comprising between 400 and 1,200 households and have a usually resident population between 1,000 and 3,000 persons (Office for National Statistics, 2021). Data were geocoded and plotted within GIS software ArcPro v2.9.5. As historic flooding data were only available for England, we restricted our sample to those residing within this country only.

### 2.2. Ethical statement

The University of Essex Ethics Committee has approved all data collection on Understanding Society main study, COVID-19 surveys and innovation panel waves, including asking consent for all data linkages except to health records. Requesting consent for health record linkage was approved at Wave 1 by the National Research Ethics Service (NRES) Oxfordshire REC A (08/H0604/124), at BHPS Wave 18 by the NRES Royal Free Hospital & Medical School (08/H0720/60) and at Wave 4 by NRES Southampton REC A (11/SC/0274). Informed consent for participation in the study was obtained, the overall mechanism for gaining consent for participation in the Study is oral. The study adheres to the Declaration of Helsinki.

### 2.3. Environmental data

Environmental data were utilised to identify individuals living in areas affected by flooding and regions experiencing changes in ambient temperature. Unlike acute heatwave exposure, this study focused on ambient temperatures, as this has been linked to mental health outcomes (Thompson et al., 2018). However, a significant research gap remains in understanding how area-specific changes in ambient temperatures at small geographical scales influence wellbeing, which is measured here. Given that extreme precipitation is a frequent outcome of severe weather events (Quoß & Rudolph, 2022), this study linked flooding events to small-area geographical units, as classified by national agencies in England, over multiple observational periods.

#### 2.3.1. Historic flood data

**Figure 1.**
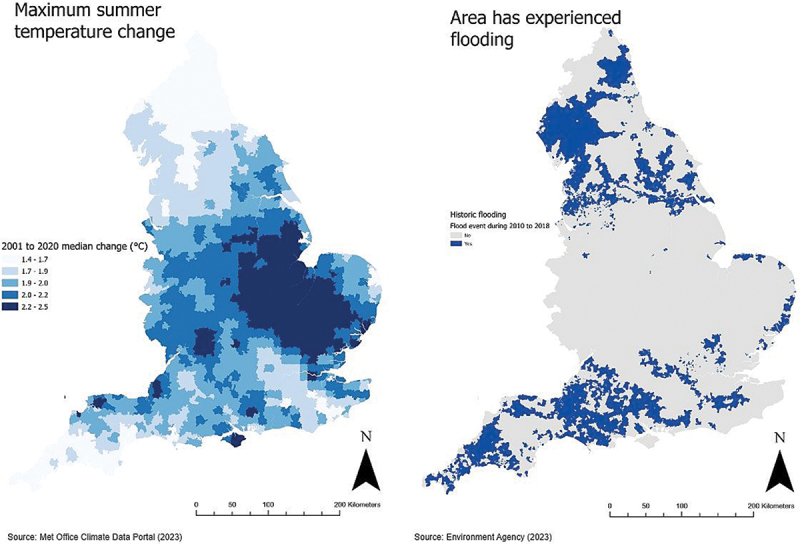
Historic flood event and climate data summarised within small-area units, England, UK.

Historic flood event data were obtained from the Environment Agency, an executive non-departmental public body of the UK Government (Environment Agency, 2023). The historic flood event data is a spatial GIS data layer containing reported and recorded flooding by local authorities from rivers, the sea, groundwater and surface water for England since 1946 and includes data on the flood event. Three flood datasets provided the year of flood event, and three flood event classifications were created to indicate whether the event occurred in the period 2010 to 2018 (shown in Figure 1), 2015 to 2018 or 2017 to 2018 to assess whether the duration of follow-up was related with the outcome measures. Flood datasets were spatially linked to LSOAs to create a binary variable of a flood event within the local area for the three time periods.

#### 2.3.2. Temperate change data

Temperature data were obtained from the Met Office, the UK’s official national weather service (Met Office Climate Data Portal, 2024), which provided the change in maximum summer temperature for the period 2001 to 2020 for the UK. This is calculated based on the highest daily maximum temperature from the summer period (June-July-August), which is then averaged to give an annual summer median maximum air temperature for small geographical areas (12km grid cells). Change in maximum summer temperature was spatially summarised within each LSOA (Figure 1) to provide the change in maximum summer temperature using the ‘Summarize Raster Within (Raster Analysis)’ tool in ArcGIS Pro which calculated the average value of all raster cells within the LSOA. Following this step, tertiles of maximum summer temperature change were calculated from 1: smallest increase to 3: largest increase for all English LSOAs.

### 2.4. Climate concern & environmental behaviour

Individual-level questions regarding climate concern and environmental behaviours were included in the study as well as two questions regarding environmental behaviours that recorded pro-environmental behaviours (via a series of 11 individual behaviour questions) and a question directly asking if they were satisfied or not with their current behaviours.

#### 2.4.1. Climate concern

Participants were asked to respond to the following statement ‘Whether, on the whole, you personally believe or do not believe people in the UK will be affected by climate change in the next 30 years’. Responses were ‘Yes, I do believe this’ or ‘No, I do not believe this’.

#### 2.4.2. Pro-environmental behaviour

Participants were asked to self-complete information on how often they engage in each of the 11 pro-environmental behaviours. They were (1) ‘Leave your TV on standby at night’, (2) ‘Switch off lights in rooms that aren’t being used’, (3) ‘Keep the tap running while you brush your teeth’, (4) ‘Put more clothes on when rather than turning on the heater’, (5) ‘Not buying something because of too much packaging’, (6) ‘Buy recycled paper products such as toilet paper or tissues’, (7) ‘Take your own shopping bag when shopping’, (8) ‘Use public transport rather than travel by car’, (9) ‘Walk or cycle for short journeys less than 2–3 miles’, (10) ‘Car share with others who need to make a similar journey’, and (11) ‘Take fewer flights’. Possible responses to these statements range from ‘1. Never’ to “5. Always, as per previous studies (Powdthavee et al., 2024; Rüttenauer, 2024), the 11 questions were averaged into an overall score.

#### 2.4.3. Satisfaction with current lifestyle and environment

Participants were asked ‘which of these best describes how you feel about your current lifestyle and the environment?’ and responses were dichotomised into the following: ‘Satisfied’ (1, I’m happy with what I do at the moment) or ‘do more’ (0, I’d like to do a bit more to help the environment, I’d like to do a lot more to help the environment).

### 2.5. Outcomes

Three wellbeing outcome variables were included:

#### 2.5.1. Psychological distress

The General Health Questionnaire (GHQ) was used to assess psychological distress, the caseness version was used for analysis that converts the 12 GHQ question responses to a single scale by recoding 1 and 2 values on individual variables to 0, and 3 and 4 values to 1, and then summing, giving a scale running from 0 (the least distressed) to 12 (the most distressed) (Cox et al., 1987).

#### 2.5.2. Life satisfaction

The measure came from the variable ‘Satisfaction with life overall’. Responses to the statement were dichotomised into a binary outcome of ‘satisfied’ (1, completely satisfied, mostly satisfied and somewhat satisfied) or ‘not satisfied’ (0, neither satisfied nor dissatisfied, somewhat dissatisfied, mostly dissatisfied and completely dissatisfied).

#### 2.5.3. Optimism

The measure came from the question ‘Please select the answer that best describes your experience of each over the last 2 weeks. I’ve been feeling optimistic about the future’. Responses to the question were dichotomised into a binary outcome of ‘optimistic’ (1, all of the time, often and some of the time) or ‘not optimistic’ (0, none of the time and rarely).

### 2.6. Statistical analysis

#### 2.6.1. Sociodemographic characteristics, environmental behaviour, and climate concern

We describe variation in the climate concern and environmental behaviour variables by the following individual-level covariates: age (16 to 24, 25 to 34, 35 to 44, 45 to 54, 55 to 64, 65+ years), sex (male, female), employment status (employed, student, unemployed, retired), material deprivation (Question: ability to keep up with bills and regular debt repayments; Responses: we have this, can’t afford it, don’t need it), education (none, aged 16 school leaver (GCSE), Aged 18 school leaver (A-level), degree or higher), self-rated health (excellent/very good/good, fair/poor), feeling lonely (hardly ever/never, sometimes/often), urban/rural status (based on residential address using the Office for National Statistics Rural and Urban Classification of Output Areas) and area-level deprivation (quintiles, from most (1) to least (5) deprived using the English Index of Multiple Deprivation (IMD)). The covariates were included based on their previous associations with likelihood of the three wellbeing outcomes and could affect climate concern and pro-environmental behaviours but would not be caused by the exposure. F-Tests in one-way ANOVA were used to examine the differences in reported environment between categorical sociodemographic variables.

#### 2.6.2. Climate concern, environmental behaviours, and wellbeing outcomes

Binary logistic regression models that allowed for a complex sample structure and weights to adjust for differential and non-responses were used to explore the associations between climate concern, pro-environmental behaviours and wellbeing outcomes (linear regression used for pro-environmental behaviour and psychological distress outcomes, bound to outcome limits). The models were performed unadjusted and adjusted for sociodemographic factors and models were performed separately for each wellbeing outcome. The pro-environmental behaviours outcome was an average score across 11 individual behaviour questions. The environmental behaviour outcome was how do you feel about your current lifestyle and the environment (‘satisfied’ vs ‘do more’).

#### 2.6.3. Heat and flood events, climate concern, environmental behaviours, and wellbeing outcomes

Residing in an area that experienced a heat and/or flood event is described by socioeconomic deprivation. Three environmental event variables were modelled within binary logistic models that allowed for a complex sample structure and weights to adjust for differential and non-responses (more detail below) were used to explore the association between environmental events, climate concern, pro-environmental behaviours, and wellbeing outcomes (linear regression used for psychological distress outcome). Environmental events were local area flooded in the previous year (yes/no), change in summer temperature between 2001 and 2020 (tertiles from smallest to largest change), and areas that had both flooded in the previous year and experienced the largest temperature increases. Sensitivity analysis was performed for time since flooding (using the following definitions: previous 3, 8 and 17 years), and found no impact on the outcomes.

All models used inverse probability weights developed and provided by Understanding Society to ensure results were representative of the population from which respondents were drawn. Weighting was applied to adjust for unequal selection and sample fraction, and to adjust for differential and non-responses using the svy command within STATA to compute finite population corrections. Analyses were further adjusted for season the participant questionnaire was administered (Spring: 25.5%; Summer: 24.1%; Autumn: 24.5%; Winter: 26.0%), government geographical region, and performed in StataMP 18.

## 3. Results

In wave 10, 27033 participants resided in England, of those 7.7% did not answer questions relating to climate concern or behaviours and were removed from the analysis. The final sample was 24,950.

### 3.1. Individual-level variation in climate concern and environmental behaviours by sociodemographic factors

#### 3.1.1. Satisfaction with environmental behaviour

Fifty-nine per cent (n: 14648) of respondents reported being satisfied with their current lifestyle and environment (Table 1). Satisfaction levels varied with age, individuals aged 65 and above exhibited a higher satisfaction rate (n: 4,361; 70%) compared to those between 16 and 24 years (n: 1,625; 53%). Disparities were also evident based on employment status, with retired individuals reporting greater satisfaction than those in education. Additionally, satisfaction levels are associated with education, with individuals holding higher qualifications reporting lower satisfaction than their counterparts with lower qualifications. Furthermore, there were differences in satisfaction based on loneliness, as individuals who reported hardly ever or never feeling lonely expressed higher satisfaction compared to their counterparts.

**Table 1.** Individual-level variation in environmental concern and behaviour by sociodemographic factors

Variables		UK affected by climate change next 30 years				Environmental behaviour (composite of actions)			How you feel about your current lifestyle and environment (subjective)			
		Yes		No		Mean			Satisfied		Do more	
		n	%	n	%	n	mean	Std. dev.	n	%	n	%
Age	16 to 24 yr	2,782	82.42	593	17.58	2837	3.113	0.536	1,800	52.6	1,625	47.5
	25 to 34 yr	2,614	80.8	621	19.2	2736	3.113	0.525	1,717	52.3	1,567	47.7
	35 to 44 yr	2,935	84.47	539	15.53	2891	3.119	0.521	1,856	53	1,646	47
	45 to 54 yr	3,594	84.11	679	15.89	3575	3.124	0.487	2,456	56.9	1,860	43.1
	55 to 64 yr	3,581	85.59	603	14.41	3524	3.117	0.518	2,457	58.3	1,758	41.7
	65+ yr	5,088	83.63	996	16.37	5392	3.124	0.511	4,361	70.3	1,845	29.7
		F = 4.52; *p* < 0.001				F = 0.67; *p* = 0.6461			F = 97.96; *p* < 0.001			
Sex	Male	9,649	81.51	2,188	18.49	10,026	3.116	0.509	7,329	61.3	4,624	38.7
	Female	10,942	85.58	1,844	14.42	10,924	3.122	0.52	7,320	56.3	5,673	43.7
		F = 67.53; *p* < 0.001				F = 0.15; *p* = 0.6973			F = 68.94; *p* < 0.001			
Employment status	Employed	11,917	84.45	2,194	15.55	11,814	3.117	0.51	7,857	55.1	6,391	44.9
	Student or training	1395	85.57	235	14.43	1,370	3.102	0.54	793	48.1	857	52
	Unemployed	1,402	76.46	431.7	23.54	1,532	3.139	0.50	1,114	59.3	764	40.7
	Retired	5,873	83.44	1,166	16.56	6,223	3.123	0.52	4,877	68.1	2,282	31.9
		F = 21.10; *p* < 0.001				F = 0.96; *p* = 0.4107			F = 127.14; *p* < 0.001			
Material deprivation	Have this	13,021	84.58	2,375	15.42	12,769	3.115	0.52	8,403	54	7,157	46
	Can’t afford it	993.7	76.53	304.7	23.47	1,032	3.164	0.53	804	60.9	516	39.1
	Don’t need it or NA	327.9	74.57	111.9	25.43	336	3.112	0.51	296	65.7	155	34.3
		F = 33.00; *p* < 0.001				F = 0.06; *p* = 0.9389			F = 7.06; *p* < 0.001			
Education	Aged 16 school leaver (GCSE)	5,154	79.34	1,342	20.66	5,334	3.132	0.51	4,393	66.5	2,213	33.5
	Aged 18 school leaver (A-level)	2,248	85.16	392	14.84	2,210	3.098	0.55	1,367	51.3	1,299	48.7
	Degree or higher	7,767	89.7	892	10.3	7,154	3.113	0.51	4,041	46.3	4,680	53.7
	None	3,483	78.29	966	21.71	4,058	3.119	0.52	3,282	72.4	1,253	27.6
		F = 126.81; *p* < 0.001				F = 1.03; *p* = 0.3764			F = 364.48; *p* < 0.001			
Self-rated health	Excellent/very good/good	16,150	84.03	3,068	15.97	16,005	3.114	0.52	11,315	58.1	8,149	41.9
	Fair/Poor	4,430	82.21	958	17.79	4,409	3.140	0.51	3,324	60.8	2,145	39.2
		F = 4.79; *p* = 0.0287				F = 5.35; *p* = 0.0208			F = 12.71; *p* < 0.001			
How often feels lonely	Hardly ever/never	12,328	83.5	2,435	16.5	12,245	3.122	0.52	9,368	62.6	5,590	37.4
	Sometimes/Often	8,217	83.86	1,582	16.14	8,106	3.115	0.52	5,231	52.7	4,689	47.3
		F = 3.24; *p* = 0.0719				F = 0.87; *p* = 0.3522			F = 301.13; *p* < 0.001			
Urban/Rural status	urban	15,899	82.67	3,333	17.33	16,314	3.118	0.51	11,573	59.4	7,923	40.6
	rural	4,695	87.04	699	12.96	4642	3.122	0.52	3,075	56.4	2,379	43.6
		F = 38.72; *p* < 0.001				F = 0.41; *p* = 0.5226			F = 9.20; *p* = 0.002			
Area deprivation	Most deprived	3,385	77.7	972	22.3	3793	3.136	0.51	3,003	67.8	1,429	32.2
	2	3,702	81.47	842	18.53	3880	3.105	0.52	2,799	60.7	1,810	39.3
	3	4,185	84.22	784	15.78	4256	3.123	0.53	2,918	57.9	2,120	42.1
	4	4,523	85.72	753	14.28	4443	3.136	0.50	2,997	56.2	2,337	43.8
	Least deprived	4,581	87.74	640	12.26	4395	3.101	0.51	2,799	53.1	2,477	47
		F = 44.15; *p* < 0.001				F = 2.77; *p* = 0.0256			F = 45.23; *p* < 0.001			
Overall		20,594	83.63	4,032	16.37	20,955	3.119	0.515	14,648	58.7	10,302	41.3

#### 3.1.2. Pro-environmental behaviour

In terms of pro-environmental behaviours score (average across 11 individual questions), there was an overall average score of 3.1, which indicates that they have altered their behaviours ‘Quite often’, where possible, due to climate concerns. There were no differences in pro-environmental behaviours between sociodemographic groups.

#### 3.1.3. Climate change concern

Eighty-four per cent of respondents reported they believed that the UK would be affected by climate change in the next 30 years. There was variation in the belief that the UK would be affected by climate change by a number of sociodemographic factors. Most pronounced were disparities by employment status (Employed 84%; Unemployed; 76%), material deprivation (have this 85%; Can’t afford this: 77%), educational level (Aged 16 school leaver 79%; Degree 90%), and area-level deprivation (most deprived 78%; least deprived 88%). There was no variation by health status or loneliness.

### 3.2. Association between climate concern, behaviour, psychological distress, life satisfaction and optimism

**Figure 2.**
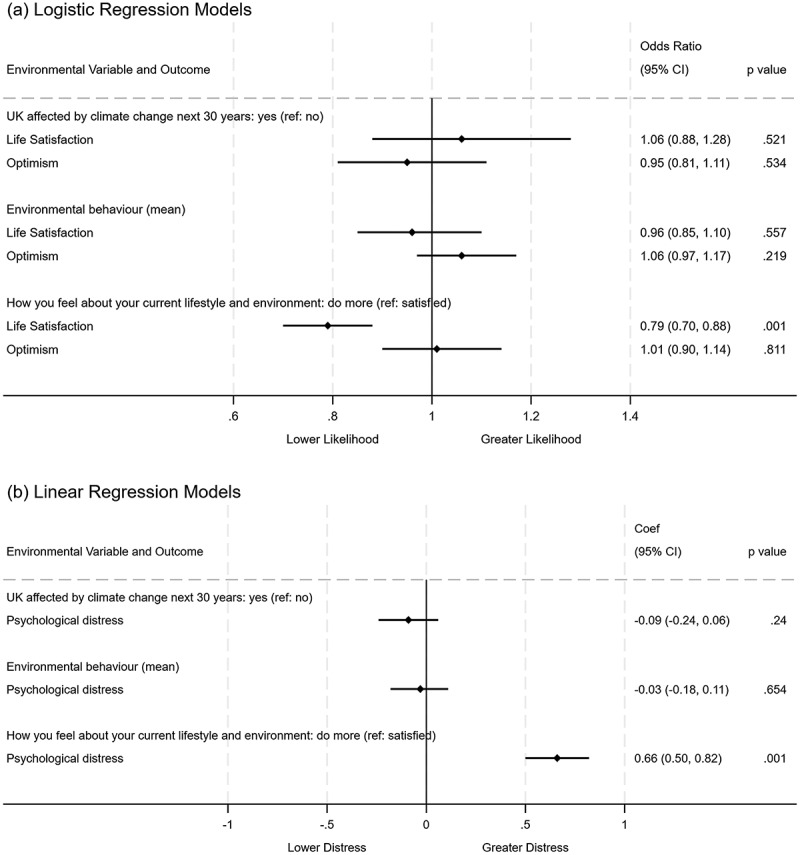
Likelihood of reporting environmental concern, pro- environmental behaviour and wellbeing outcomes.

Figure 2 describes the association between self-reported climate concern, pro-environmental behaviour and wellbeing outcomes (psychological distress, life satisfaction and optimism). The results showed that pro-environmental behaviours were not associated with any wellbeing outcomes. However, acknowledging the potential for improvement in one’s current lifestyle and environment was associated with psychological distress (Coef: 0.66; 95% CI: 0.50 to 0.82) and reduced life satisfaction (OR: 0.79; 95% CI: 0.70 to 0.88) (Figure 2).

Adjusted model for sociodemographic covariates, unadjusted model presented in supplementary Figure S1. P values stated as 0.001 in chart are <0.001; 95%CI: 95% confidence intervals; ref: model reference category.

### 3.3. Experience of climate change (residing in a flood effected or temperature changing area) by socioeconomic position and wellbeing outcomes

A total of 22,918 participants had valid geographical locations linked to spatial data and their local area socioeconomic position recorded (Table 2). A small number of participants (n = 162) resided in an area that had a recorded flood in the previous year; a marginally higher proportion in the most deprived (0.68%) compared to the least deprived (0.25%), however these numbers are too small to compute statistical differences. On the other hand, a greater proportion of participants from the least deprived areas (37%) resided in an area that experienced the largest summer temperate increase than the most deprived (25%). Only 18 participants resided in an area that experienced both the largest summer temperate increase and had flooded in the past year.

**Table 2.** Recorded local area flood events and temperature change by socioeconomic position

Area-level socioeconomic position	N	Local area flooded in the previous year (yes)		Change in summer temperature (2001 to 2019): Largest tertile		Largest heat increase and recently flooded	
		N	%	N	%	N	%
Most deprived	4090	28	0.68	989	24.18	0	0.00
2	4281	39	0.91	1215	28.39	1	0.02
3	466	41	0.89	1686	36.14	7	0.14
4	4958	42	0.85	1892	38.16	6	0.11
Least deprived	4920	12	0.25	1826	37.12	4	0.07
Total	22918	162	0.25	7609	33.20	18	0.07

**Figure 3.**
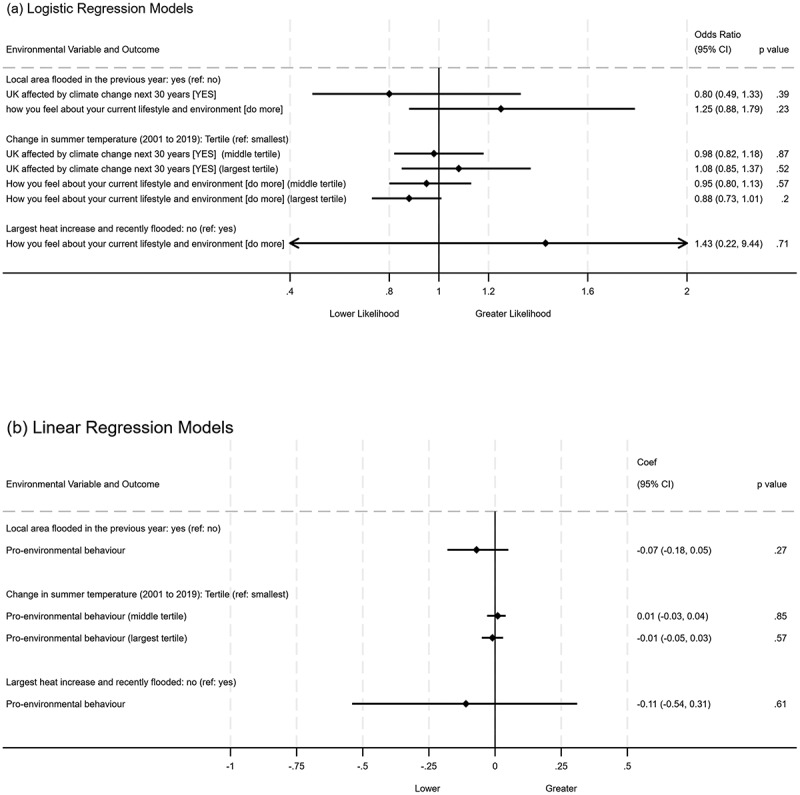
Reported environmental concern and behaviour by recorded local area flood events in the past year and temperature change.

**Figure 4.**
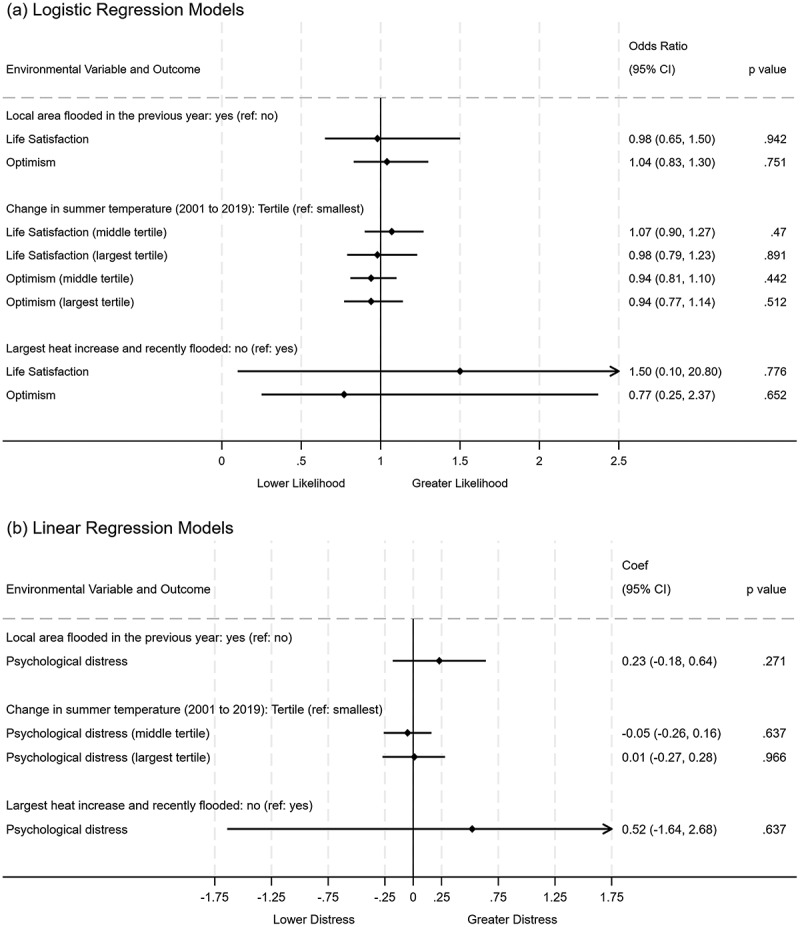
Likelihood of wellbeing by recorded local area flood events in the past year and temperature change.

Figure 3 presents the results of a model exploring whether residence in an area that either (i) suffered flooding in the past year (2017/18), (ii) underwent the largest temperature change from 2001 to 2020, or experienced both (i) and (ii) is associated with individual-level reported climate concerns or pro-environmental behaviours. Figure 4 presents the results of a model exploring the association between wellbeing outcomes by recorded local area flood events in the past year and temperature change. The results from both analyses showed no association with area-level flooding or temperature change climate concerns or behaviours, and reported psychological distress, life satisfaction, or optimism (Figures 3 & 4).

Adjusted model for sociodemographic covariates, unadjusted model presented in supplementary Figure S2. 95%CI: 95% confidence intervals; ref: model reference category.

Adjusted model for sociodemographic covariates, unadjusted model presented in supplementary Figure S3. 95%CI: 95% confidence intervals; ref: model reference category.

## 4. Discussion

### 4.1 summary of results

The aims of the study were to explore whether climate concerns and pro-environmental behaviours (satisfaction and self-reported behaviours) vary by individual-level sociodemographic factors, and whether these were associated with wellbeing outcomes, including psychological distress, life satisfaction, and optimism. Environmental data were spatially linked to identify whether participants resided in an area that had experienced a flood event or their summer temperature change, and if this was associated with climate concerns, environmental behaviours, and wellbeing outcomes. We found that climate concern and pro-environmental behaviour varied by sociodemographic factors. Reporting satisfaction with current lifestyle and the environment, pro-environmental behaviours across 11 individual questions, and climate concern were most frequently higher for males, older individuals, retirees, those with lower educational attainment, the unemployed, individuals who were divorced, separated, or widowed, and individuals residing in the most deprived areas. There was no association between pro-environmental behaviours, psychological distress, life satisfaction and optimism, whereas indicating this could be improved was associated with worse psychological distress and a reduced likelihood of life satisfaction.

We found that residing in an area that had experienced a flood event, underwent the largest summer temperature change, or experienced both were not associated with our wellbeing outcomes. However, only a small number of our sample experienced these events.

### 4.2. Comparison with other literature

We did not find an association between pro-environmental behaviours (measured across 11 individual actions), psychological distress, life satisfaction or optimism, despite this relationship being observed in a number of studies across diverse geographical contexts, spanning multiple continents (Capstick et al., 2022). A meta-analysis of 78 studies revealed an overall connection between reporting pro-environmental behaviours and subjective wellbeing, with a stronger effect size observed when both measures were precisely collected and various types of pro-environmental behaviours were reported (Zawadzki et al., 2020). Our study extended beyond a single subjective measure to accurately capture the frequency of engagement in a number of pro-environmental actions, such as the proportion of paper recycled, or journeys taken on foot or cycled rather than by motorised modes (Gatersleben, 2018). In addition to pro-environmental behaviours measured across 11 individual actions, we were also able to assess satisfaction with environmental lifestyle, which found this was associated with wellbeing. Our analysis revealed that poor satisfaction was linked to lower life satisfaction and increased psychological distress. This suggests that wellbeing is influenced not only by engaging in pro-environmental behaviours but also by the perceived adequacy or satisfaction derived from those actions. These findings underscore the importance of adopting a multidimensional approach to understanding pro-environmental behaviours—considering both the behaviours themselves and the satisfaction associated with them (Ertz & Sarigöllü, 2019). Moreover, self-identifying as someone who performs a satisfactory level of pro-environmental actions may help reduce environmental guilt, potentially mitigating pathways to negative wellbeing outcomes (Lacasse, 2016). By exploring these nuanced relationships, we highlight the need to consider both behavioural engagement and personal satisfaction in efforts to understand and improve wellbeing in the context of climate concerns.

Addressing climate change requires behaviour change (Stapleton et al., 2022). In their review of behaviour change models and climate action, Whitmarsh et al. (2021) emphasise that behaviour change is a crucial component in tackling the climate crisis. However, they also identify limitations and gaps in existing models that may impede meaningful progress. Consistent with our findings, Whitmarsh et al. highlight that sociodemographic factors, such as income and employment, play a more significant role than psychological factors in shaping climate-friendly behaviours. Additionally, they discuss other multidimensional influences, including social norms, spillover effects, collective action, and the interplay of capability, opportunity, and motivation. Both Whitmarsh et al. (2021) and Stapleton et al., (2022) review various behaviour change models, alongside their critiques, to inform the development of more effective interventions. Our study contributes to this field by providing evidence that pro-environmental behaviour is positively associated with wellbeing, supporting the design of interventions that encourage sustainable actions while promoting individual and societal benefits.

We did not find an association between greater summer temperatures in the UK and wellbeing impacts. This may be due to the UK not experiencing marked increases in summer temperature that are associated with poorer wellbeing outcomes, when compared to other regions such as mid-latitude, semi-arid regions. Evidence from warmer climates may suggest a temperature threshold effect for an impact on wellbeing. For example, evidence from North America found that increased average ambient temperature above 32°C significantly reduced emotional wellbeing (Noelke et al., 2016). An Australian study found that average temperatures below 4°C and above 32°C were negatively associated with general health (Hailemariam et al., 2023). Furthermore, the mechanisms through which climate change anxiety affects wellbeing are often described as ‘temporally distal’, reflecting the delayed impact on health and wellbeing that may unfold over years or even decades. These pathways are also characterised as ‘spatially distal’, as environmental impacts often occur in distant locations or are anticipated to happen elsewhere in the future (Morris et al., 2017). This may be an important factor in contexts such as England that may not yet be experiencing such high temperatures. However, it is important to model and predict future wellbeing burdens linked to adverse weather and develop preventative mitigation strategies for this. For example, projections suggest the UK may experience increased summer temperatures between 3.8°C and 6.8°C by 2070, which will be greater in southern England and may impact wellbeing outcomes (Met Office, 2021).

Similar to our study, Anneser et al. (2024) showed that environmental exposures were not associated with wellbeing outcomes for a North American adult sample. However, they found that increased reporting of climate stress/anxiety was positively associated with increased civic engagement, such as canvassing, personal efficacy, group efficacy and voter preference. Providing a suggestion that climate concern may promote pro-environmental behaviours. We explored whether experience of a flood or heat event was patterned by socioeconomic position, however we were limited by a small sample who has experienced a flood event. Previous research found that wellbeing impacts of flooding may be greater if an individual was directly affected by these adverse events, for example if their property experienced or was at high risk of flooding. Qualitative research from individuals residing in the UK who did experience a flood event to their property found poorer wellbeing outcomes, which returned to ‘normal’ levels over time (Walker-Springett et al., 2017). Reporting of returning to ‘normal’ wellbeing levels was influenced by external factors, such as lack of agency, community connections and relationships. Twiddy et al. (2022) found that the wellbeing impacts of being directly affected by flooding ebbed and flowed were mostly concerned with future events and peaked during adverse weather forecasts for rain. Similarly, the acute impact of heatwaves on hospital admissions is found immediately after the event and reduces over time (Mastrangelo et al., 2007; Thompson et al., 2023). Community cohesion, empowerment and collective and collaborative activities have repeatedly been shown to provide some mitigation against the impact of flood events on wellbeing (Anneser et al., 2024; Walker-Springett et al., 2017; Woodhall-Melnik & Grogan, 2019). This highlights the importance of bottom-up community action and involvement in developing local flood mitigation strategies that may provide some protection against adverse wellbeing impacts. Indeed, sustainable and pro-environmental behaviours and engagement can support efforts mitigating the psychosocial impact of the climate crisis (Hayes et al., 2018).

The negative consequences of climate change worry may accumulate over time, for example evidence from New Zealand found that climate worry was associated with increased psychological distress one year later but not life satisfaction (McBride et al., 2021). We recommend future longitudinal research to monitor the impact of climate events on wellbeing. For example, applying rapid surveys in areas that experience extreme weather events to understand the immediate impact of heat and flood events (both during and after their occurrence) on wellbeing that are linked to existing cohorts to examine changes following an event in the short, medium and long term. As well as understanding the spatial proximity to these events and to measure the geographic dispersal effects on wellbeing outcomes.

### 4.3. Strengths and limitations

Our study included a number of strengths. We used high-quality and precise spatial data from two reputable UK government agencies, namely the Met Office and Environment Agency, to compile information on heat and flooding events and spatially aggregate within small geographic areas across England. Capturing the date of the flood allowed us to conduct a sensitivity analysis spanning multiple years, enabling the measurement of the short- and medium-term impact of such events. Additionally, we employed a large, nationally representative cohort that included inquiries about climate concern, behaviours and wellbeing, alongside numerous sociodemographic variables for comprehensive analysis. We were able to adjust our analysis for season of questionnaire administered to account for varying temperatures.

While flooding events primarily impact the wellbeing of individuals whose homes and lives face significant disruption, our study was limited in its ability to determine whether participants directly experienced a flood event or assess the severity of such occurrences. Future research could benefit from including questions about personal flood experiences (e.g. timing and context) or incorporating residential location data for precise spatial proximity analysis. This approach, coupled with information on flood depth and prior warnings, would allow for a more robust assessment of mental health and wellbeing impacts. Additionally, several factors—such as levels of climate adaptation, connection to nature, and social support—may influence wellbeing outcomes and pro-environmental behaviours. These elements should be prioritised in future studies to provide a more comprehensive understanding of the interplay between environmental events and human responses (Ma et al., 2022; Martin et al., 2020; Quinn et al., 2023; Thomas et al., 2014).

Our measurements were constrained in terms of pro-environmental behaviours, satisfaction with environmental, and specific wellbeing outcomes associated with climate. We were unable to directly establish whether climate concern was linked to wellbeing. Recognising established pathways between climate concern and wellbeing, future studies should include specific questions addressing wellbeing and climate, with particular emphasis on young people, given the widespread prevalence of climate change-related worry and concern in children and youth globally (Hickman et al., 2021). Our question asked respondents to report their perception of current environmental behaviours, and this does not mean they are engaging in environmentally friendly behaviours, reiterating our previous call for future research to accurately capture engagement in environmentally friendly behaviours.

## 5. Conclusions

In conclusion, reporting of climate concern and pro-environmental behaviours varied by sociodemographic factors, typically those of older age and in lower socioeconomic groups were most satisfied with their current behaviours. Those who were most satisfied with their current behaviours and the environment reported greater wellbeing outcomes, however engagement in pro-environmental behaviours was not associated with wellbeing outcomes. We highlight that for English residents, residing in an area that had experienced a flood event, underwent the most significant temperature change, or both, was not associated with wellbeing outcomes measured in this study. Further research is urgently needed to investigate the direct impact of climate events on wellbeing. Existing studies are limited by small sample sizes and yield inconclusive or mixed findings, highlighting the need for more robust and comprehensive analyses.

Our results highlight variation in perceptions of the climate crisis and pro-environmental behaviours by sociodemographic factors. Regardless, perceiving satisfaction with pro-environmental behaviours was associated with higher wellbeing and could act to mitigate against the potential adverse effects of eco-anxiety.

## Supplementary Material

Sup_Fig.1.png

Sup_Fig.2.png

Sup_Fig.3.png

## Data access statement

Understanding Society data are available via the UK Data Service, further information via data holders: University of Essex, Institute for Social and Economic Research (2024). Understanding Society: Waves 1–14, 2009–2023 and Harmonised BHPS: Waves 1–18, 1991–2009. [data collection]. 19th Edition. UK Data Service. SN: 6614, http://doi.org/10.5255/UKDA-SN-6614-20.

## Rights retention statement

For the purpose of open access, the author(s) has applied a Creative Commons Attribution (CC BY) licence to any Author Accepted Manuscript version arising from this submission.
