# Supplementary figures and images for: Linkages between socioeconomic inequalities, pro-environmental behaviours, climate change concerns and experiences, and wellbeing outcomes in England
Source: Sustain Environ. 2025 May 12;11(1):2500182. doi: 10.1080/27658511.2025.2500182 (PMC12306679; doi:10.1080/27658511.2025.2500182)

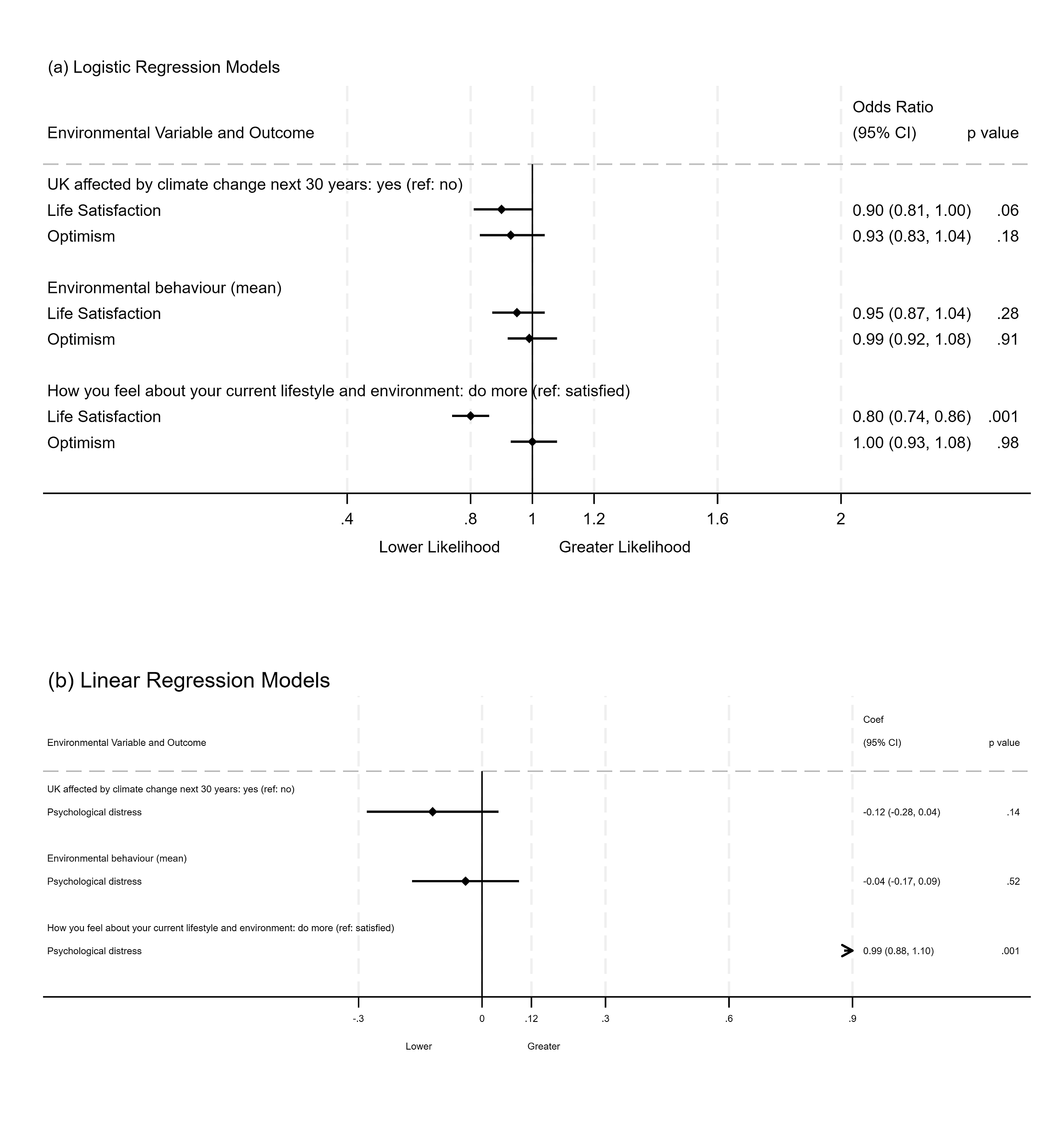

Supplement: Sup_Fig.1.png [file OAES_A_2500182_SM8482.png]

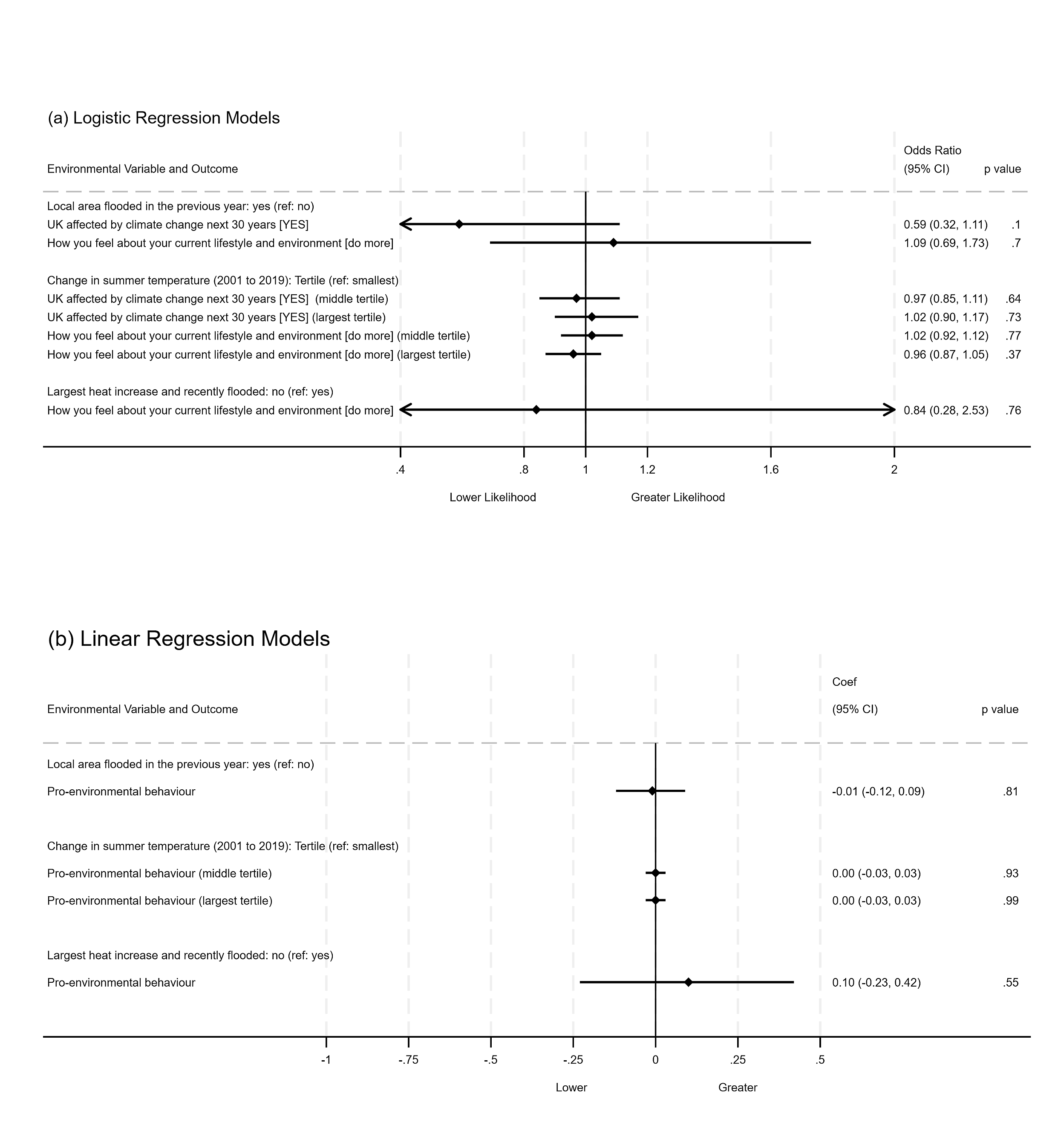

Supplement: Sup_Fig.2.png [file OAES_A_2500182_SM8481.png]

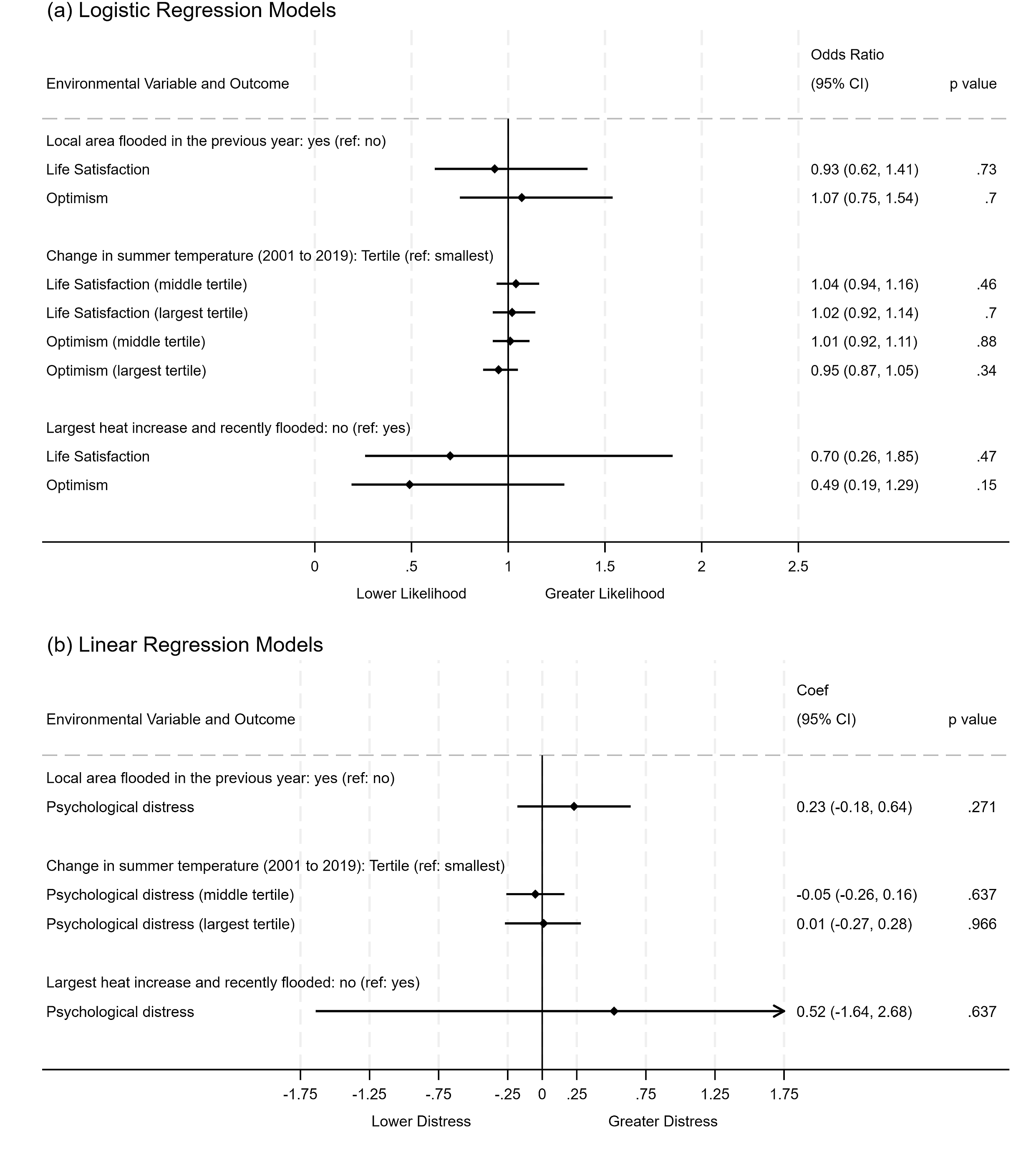

Supplement: Sup_Fig.3.png [file OAES_A_2500182_SM8480.png]
